# Affective instability and impulsivity predict nonsuicidal self-injury in the general population: a longitudinal analysis

**DOI:** 10.1186/s40479-016-0051-3

**Published:** 2016-12-13

**Authors:** Evyn M. Peters, Marilyn Baetz, Steven Marwaha, Lloyd Balbuena, Rudy Bowen

**Affiliations:** 1 Department of Psychiatry, University of Saskatchewan, Saskatoon, SK S7N0W8 Canada; 2 Division of Mental Health and Wellbeing, Warwick Medical School, University of Warwick, Coventry, UK; 3 Mood and Anxiety Disorders Service, Caludon Centre, Coventry, UK

**Keywords:** Borderline personality, Nonsuicidal self-injury, Affective instability, Impulsivity, Mood instability, Emotional dysregulation

## Abstract

**Background:**

Impulsivity and affective instability are related traits known to be associated with nonsuicidal self-injury, although few longitudinal studies have examined this relationship. The purpose of this study was to determine if impulsivity and affective instability predict future nonsuicidal self-injury in the general population while accounting for the overlap between these traits.

**Methods:**

Logistic regression analyses were conducted on data from 2344 participants who completed an 18-month follow-up of the 2000 British National Psychiatric Morbidity Survey. Affective instability and impulsivity were assessed at baseline with the Structured Clinical Interview for DSM-IV Axis II Personality Disorders. Nonsuicidal self-injury was assessed at baseline and follow-up during semi-structured interviews.

**Results:**

Affective instability and impulsivity predicted the onset of nonsuicidal self-injury during the follow-up period. Affective instability, but not impulsivity, predicted the continuation of nonsuicidal self-injury during the follow-up period. Affective instability accounted for part of the relationship between impulsivity and nonsuicidal self-injury.

**Conclusions:**

Affective instability and impulsivity are important predictors of nonsuicidal self-injury in the general population. It may be more useful to target affective instability over impulsivity for the treatment of nonsuicidal self-injury.

## Background

Nonsuicidal self-injury (NSSI) refers to socially unacceptable damage to one’s body (e.g., skin-cutting, burning) without suicidal intent [1, 2]. Aside from directly causing bodily harm, NSSI is a personal and public health problem, and may indicate severe psychopathology [1]. Usually beginning in early adolescence, it can have a chronic course and evolve into suicidal thoughts and behaviour over time [1–3]. The behaviour is insidious because the majority of people engaging in NSSI avoid seeking clinical attention [1].

NSSI is theorized to be a maladaptive emotional regulation strategy because an attempt to alleviate negative feelings is a common motive endorsed by people who self-injure [4–8]. NSSI is most strongly associated with variability in negative affect rather than stable negative affect [8–10]. Affective instability, defined as the tendency to experience rapid and intense mood swings that are difficult to control, occurs in patients suffering from a variety of psychiatric disorders as well as in the general population [11]. Not surprisingly, a number of studies have shown that affective instability is associated with NSSI in clinical and nonclinical populations [8–10, 12].

One model has expanded upon the affect regulation theory of NSSI [4, 5, 7] by proposing that NSSI is performed to distract individuals from reciprocal cycles of negative affect and rumination called emotional cascades [8, 13]. Emotional cascades and affective instability are similar concepts and may only differ in that the former, by definition, involves cognitively ruminating upon negative affect [8, 13]. Interestingly, one study found that unstable rumination interacted with unstable negative affect to predict NSSI, and that negative affect instability and rumination instability were highly correlated [8].

NSSI is also associated with impulsivity [5, 14–16], the tendency to act suddenly without concern for negative consequences [17]. People who engage in NSSI tend to score higher on impulsivity questionnaires but, paradoxically, often do not display impulsive response styles on laboratory tests [15]. One possible reason is that people who self-injure tend to behave impulsively while experiencing negative affect, a phenomenon sometimes referred to as negative urgency [17], and this contextual factor is not accounted for by laboratory measures of impulsivity [15]. These findings were supported by a recent meta-analysis, although the authors note there is a paucity of longitudinal research addressing the relationship between impulsivity and NSSI [16]. Past research has also recommended that future studies examine how impulsivity overlaps with negative affect in relation to NSSI [15].

It has been suggested that impulsive individuals may be more likely to choose self-injury as an emotional regulation strategy when they experience strong, poorly controlled emotional states [5, 15]. On the other hand, emotional dysregulation contributes to impulsivity [13, 17, 18], and affective instability has been shown to predict impulsive behaviours even after controlling for trait impulsivity [18]. Therefore, impulsivity may be only part of the mechanism associated with self-injurious acts, given that people who experience affective instability are more likely to behave impulsively in general, as well as harming themselves to control mood swings.

The purpose of this study was to investigate the impact of impulsivity and affective instability on NSSI using a longitudinal design. Specifically, we sought to determine if impulsivity and affective instability could predict future episodes of NSSI in a general population sample while also accounting for the overlap between these traits. Based on a theoretical model in which affective instability contributes to both NSSI [1, 8–10, 12] and impulsivity in general [13, 17, 18], we hypothesized that the association of impulsivity with NSSI would be attenuated when affective instability was controlled.

## Methods

### Sample

The sample included participants from the 2000 British National Psychiatric Morbidity Survey (NPMS [19, 20]). The NPMS was a national survey designed to study mental illness and related variables among participants aged 16 to 74 living in private residences in England, Scotland, and Wales [20]. We also used an 18-month follow-up survey in which a subsample of the 2000 survey respondents underwent repeat assessment procedures [21]. For both surveys, data was gathered through face-to-face structured interviews and self-report questionnaires [20, 21]. Of the 8580 participants who completed the first survey, those eligible for the follow-up survey were sampled from three subgroups: (1) individuals with a common mental disorder (*n* = 1685), defined as a score of 12 or higher on the revised Clinical Interview Schedule (CIS-R [22]); (2) individuals without a common mental disorder but with multiple symptoms (*n* = 1032), defined as CIS-R scores between 6 and 11; and (3) individuals without a mental disorder and few symptoms (*n* = 819), defined as CIS-R scores between 0 and 5 [21]. Therefore, participants eligible to take part in the follow-up survey were likely to have a mental disorder or associated symptoms. Of these participants, 3045 were contacted and 2406 were interviewed [21]. Participants lost to follow-up were likely to be younger, single, have lower socioeconomic status, and to have smoked cigarettes or have used illicit drugs in the past year [21].

### Material and measures

Impulsivity and affective instability were measured at baseline (Time 1) within the borderline section of the Structured Clinical Interview for DSM-IV Axis II Personality Disorders (SCID-II [23]). The SCID-II assesses for these traits with the questions “Have you often done things impulsively?” and “Do you have a lot of sudden mood changes?” [23]. These questions were designed to query long-standing patterns of affective instability and impulsive behaviour as criteria for the diagnosis of borderline personality disorder [1]. Responses were coded as 1 (“Yes”) or 0 (“No”).

NSSI at baseline (Time 1) was assessed by semi-structured interviews with the CIS-R question ‘Have you ever deliberately harmed yourself but not with the intention of killing yourself?’ [20]. NSSI during the 18-month follow-up period was assessed at Time 2 with a modified version of the same question that asked specifically about NSSI since the Time 1 survey [21]. Both NSSI variables (Time 1 and Time 2) were coded as 1 (present) or 0 (not present).

Participant age and sex were included as covariates. This was done because younger age is associated with affective instability [24], impulsivity [25], and NSSI [26], and to control for potential sex differences in affective instability [27] and impulsivity [28]. In addition, we also controlled for whether participants met the DSM-IV diagnostic criteria for borderline personality disorder. This was done to account for the possibility that participants endorsing impulsivity and affective instability were also suffering from borderline personality disorder. To establish this diagnosis, participants first completed a self-report version of the SCID-II [20]. A probability sampling framework based on the total number of SCID-II borderline criteria endorsed in the self-report segment of the survey was then used to reselect participants to be reassessed by trained interviewers using the SCID-II [20]. The sampling scheme was designed in such a way that participants who endorsed more borderline traits on the self-report SCID-II were more likely to be selected for reassessment [20]. Participants who met the DSM-IV diagnostic criteria as determined during clinical interviews with the SCID-II were considered to have the disorder. The variable representing this diagnosis was coded as 1 (present) or 0 (not present).

### Analysis

Simple logistic regression analyses were used to examine for age and sex differences between participants with and without affective instability and impulsivity. Multiple logistic regression analyses were used to predict future episodes of NSSI during the 18-month follow-up period. Impulsivity and affective instability were first included separately as predictors, and each model was tested with and without covariates included. Analyses were also restricted to include only participants with or without a history of NSSI at Time 1, making the results relevant to the continuation or development of NSSI during the 18-month follow-up period, respectively. We conducted four additional multiple logistic regression analyses predicting NSSI during the 18-month follow-up period to test for interactions between affective instability and age, affective instability and sex, impulsivity and age, and impulsivity and sex. The association between affective instability and impulsivity was then established with simple logistic regression, and a final exploratory regression model was tested in which affective instability and impulsivity were included simultaneously as predictors of NSSI. We also allowed for an interaction between impulsivity and affective instability to determine if each trait had a unique contribution to NSSI.

All statistical analyses were conducted with Stata [29] using probability weights. Probability weights accounted for (1) the probability of selection from different areas of the country and from different sized households, (2) baseline survey response rates by region, age, and sex, (3) the probability of being selected to participate in the follow-up survey, and (4) differential follow-up survey response rates between participant subgroups identified with a decision tree analysis [21]. Descriptive statistics and logistic regression analyses were conducted using survey estimation commands that also took into account the multi-stage stratified design of the NPMS. A subpopulation command was used to restrict the calculation of weighted estimates to include data from the participants who had complete data for all the study variables (*n* = 2344). Standard errors were calculated using Taylor linearization.

## Results

The mean age of the final sample (*n* = 2344) was 44.6 years (weighted mean = 43.4 years, linearized *SE* = 0.50) and 57.5% were female (weighted % female = 50.6, linearized *SE* = .016). The number of participants reporting a history of NSSI at Time 1 was 85 (weighted % of sample = 2.36, linearized *SE* = .003; weighted mean age = 30.4 years, linearized *SE* = 1.48; weighted % female = 60.2, linearized *SE* = .067). The number of participants at Time 2 who reported having engaged in NSSI during the 18-month follow-up period was 23 (weighted % of sample = 0.61, linearized *SE* = .002; weighted mean age = 27.0 years, linearized *SE* = 2.29; weighted % female = 43.9, linearized *SE* = .139). Of these participants, 12 (weighted % of sample = 0.33, linearized *SE* = .001; weighted mean age = 27.2 years, linearized *SE* = 3.41; weighted % female = 33.1, linearized *SE* = .187) had no previous history of NSSI. Overall, the number of participants who reported a history of NSSI at either Time 1 or Time 2 was 97 (weighted % of sample = 2.69, linearized *SE* = .003). Descriptive statistics for participants with and without a history of NSSI at any time are presented in Table 1. Affective instability was associated with being female (*OR* = 1.60, linearized *SE* = 0.24, *p* = .001) and younger age (*OR* = 0.97, linearized *SE* = .005, *p* < .001). Impulsivity was not associated with sex (*OR* = 1.11, linearized *SE* = 0.13, *p* = .40) or age (*OR* = 1.00, linearized *SE* = .004, *p* = .57).

**Table 1:** Descriptive statistics for participants with and without a lifetime history of NSSI

Variables	Unweighted *n* or *M* (and weighted estimates)		*OR* (*SE*)
	No NSSI (*n* = 2247)	NSSI (*n* = 97)	
SCID-II			
AI	455 (12.9%, *SE* = .008)	55 (49.9%, *SE* = .063)	6.72 (1.76)***
Impulsivity	1071 (41.2%, *SE* = .015)	62 (63.7%, *SE* = .064)	2.50 (0.71)**
BPD	3 (0.05%, *SE* = .0003)	5 (2.58%, *SE* = .014)	53.6 (43.8)***
Demographics			
Age	45.1 (43.7, *SE* = 0.52)	34.1 (30.0, *SE* = 1.34)	0.93 (.009)***
Female	1287 (50.4%, *SE* = .016)	60 (56.9%, *SE* = .062)	1.30 (0.33)

*Note*. Odds ratios (and linearized standard errors) are from weighted simple logistic regression analyses with each variable predicting lifetime NSSI

*NSSI* nonsuicidal self-injury, *SCID-II* Structured Clinical Interview for DSM-IV Axis II Personality Disorders, *AI* affective instability, *BPD* borderline personality disorder

*p < .05

**p < .01

***p < .001

Results from the multiple logistic regression analyses predicting NSSI are presented in Table 2. As can be seen, affective instability and impulsivity predicted the onset of NSSI, whereas only affective instability predicted the continuation of NSSI. This pattern of results remained with age, sex, and borderline diagnosis included as covariates. Nonsignificant interactions were found between affective instability and age (*OR* = 1.03, linearized *SE* = 0.03, *p* = .34), affective instability and sex (*OR* = 0.34, linearized *SE* = 0.36, *p* = .32), and impulsivity and age (*OR* = 0.99, linearized *SE* = 0.04, *p* = .72). There was a significant interaction between impulsivity and sex (*OR* = 0.06, linearized *SE* = 0.08, *p* = .03), such that impulsivity among females was inversely related to NSSI. In this model the main effect of impulsivity remained significant (*OR* = 39.2, linearized *SE* = 42.3, *p* = .001), whereas the main effect of sex was nonsignificant (*OR* = 7.70, linearized *SE* = 8.74, *p* = .07).

**Table 2:** Multiple logistic regression analyses predicting NSSI during the 18-month follow-up period

Predictors	Any NSSI	Development of NSSI	Continuation of NSSI
Univariate:			
Impulsivity	6.62 (3.73)**	11.7 (9.85)**	2.13 (1.78)
AI	24.1 (13.0)***	16.7 (11.8)***	10.4 (9.32)**
Multivariate:			
Impulsivity	6.39 (3.83)**	11.1 (9.67)**	2.47 (2.74)
BPD	155.6 (164.3)***	87.3 (143.7)**	18.2 (15.9)**
Age	0.91 (0.02)***	0.91 (0.03)**	0.95 (0.03)
Sex	0.77 (0.41)	0.50 (0.38)	0.79 (0.87)
Multivariate:			
AI	17.3 (10.5)***	13.5 (10.1)**	10.3 (10.6)*
BPD	38.0 (31.4)***	34.6 (45.0)**	8.59 (8.60)*
Age	0.92 (0.17)***	0.92 (0.02)**	0.95 (0.03)
Sex	0.45 (0.27)	0.30 (0.25)	0.74 (0.72)

*Note*. Values are odds ratios (and linearized standard errors). All predictors were measured at Time 1. The dependent variable in each model was the presence of NSSI during the follow-up period assessed at Time 2. Models predicting any NSSI included all participants regardless of whether they had a history of NSSI at Time 1 (*n* = 2344). Models predicting the development of NSSI included only participants with no history of NSSI at Time 1 (*n* = 2259). Models predicting the continuation of NSSI included only participants with a history of NSSI at Time 1 (*n* = 85)

*NSSI* nonsuicidal self-injury, *AI* affective instability; *BPD* borderline personality disorder

*p < .05

**p < .01

***p < .001

Affective instability and impulsivity were associated (*OR* = 2.35, linearized *SE* = 0.35, *p* < .001); 61.4% of the participants who reported affective instability reported impulsivity as well. In the final regression model that included both traits simultaneously as predictors, the effect of affective instability on NSSI remained significant (*OR* = 34.5, linearized *SE* = 39.3, *p* = .002), whereas the effect of impulsivity became nonsignificant (*OR* = 7.12, linearized *SE* = 7.89, *p* = .08). The interaction between affective instability and impulsivity was nonsignificant (*OR* = 0.46, linearized *SE* = 0.60, *p* = .55).

## Discussion

The main finding of this study is that affective instability and impulsivity predicted future NSSI in a general population sample. Both traits predicted the onset of NSSI in participants without a previous history of NSSI, whereas only affective instability predicted the continuation of NSSI in participants who had previously self-harmed. The results remained significant after controlling for age, sex, and a diagnosis of borderline personality disorder. However, the association between impulsivity and NSSI became nonsignificant after controlling for affective instability. We interpret this as evidence that part of the relationship between impulsivity and NSSI occurs because affective instability contributes to both NSSI and impulsivity in general.

Our results are partially consistent with those reported by You, Leung, Lai, and Fu [12], who found that, in a sample of Hong Kong secondary school students, impulsivity and affective instability predicted the onset of NSSI, whereas lower levels of affective instability, but not impulsivity, predicted the discontinuation of NSSI. Our results extended this research by showing that affective instability and impulsivity also predict future NSSI in the general adult population independently of borderline personality disorder. In contrast to our results, however, You et al. [12] found that impulsivity remained predictive of, and was more strongly associated with NSSI than affective instability when both traits were included in the same regression model. This discrepancy could have resulted from impulsivity being more prominent in adolescents [25], or from differences between Asian and Caucasian participants [30]. You et al. [12] also measured impulsivity with a questionnaire that lists pathological impulsive behaviours known to result from dysregulated emotions (e.g., binge eating, substance abuse, anger outbursts [13, 17, 18]), whereas we used an item that assesses the tendency to behave impulsively in general. The fact that the former was strongly associated with NSSI, and that the latter was not significantly associated with NSSI after controlling for affective instability, is consistent with research showing that emotionally driven impulsivity is the most relevant to NSSI [15, 16], and also highlights the emotional dysregulation underlying NSSI [4–10] and other maladaptive impulsive behaviours [13, 17, 18].

The nature of the relationship between impulsivity and affective instability leading to NSSI is difficult to interpret because both traits were measured over the same time period. It is possible that people who experience affective instability engage in impulsive behaviours (e.g., substance use, binge eating), in addition to NSSI, as a method of emotional regulation to cope with mood swings. This interpretation is hypothetical but consistent with theory and research concerned with the role of dysregulated emotions in impulsive behaviour [13, 17, 18]. A competing explanation is that impulsivity somehow leads to affective instability which then predisposes toward NSSI. It has been suggested that impulsive people may be more likely to engage in NSSI as an emotional regulation strategy [5, 15]. Our finding that affective instability predicted NSSI with impulsivity included in the same regression model suggests that affective instability contributes to NSSI independently of impulsivity. It seems clear that affective instability is an important predictor of NSSI in the general population.

The relationship between impulsivity and affective instability in this study is also difficult to interpret because we used a unidimensional impulsivity measure. Research has shown there are multiple dimensions of impulsivity, each correlating with a unique set of personality traits [17] and clinical variables [31]. Research using multidimensional impulsivity questionnaires has shown that negative urgency, the tendency to behave impulsively in response to negative affect [17], is the dimension of impulsivity most strongly associated with NSSI [15, 16]. It would be useful for future research to determine if negative urgency mediates or moderates the relationship between affective instability and NSSI.

It is worth mentioning that, consistent with previous research [2, 26], having a diagnosis of borderline personality disorder predicted NSSI in all models. Age is a known correlate of NSSI [1, 2, 26] that did not predict the continuation of NSSI in our study (although the odds ratios were only marginally nonsignificant, *p* < .1, and not substantially smaller than the odds ratios that reached statistical significance). Neither affective instability nor impulsivity interacted with age to predict NSSI. Consistent with previous research [2, 26], although disparate from lay opinions of NSSI, sex was not a significant predictor in any model. There was, however, a significant interaction between impulsivity and sex, such that impulsivity among females was negatively associated with NSSI. It is possible that impulsivity may be more relevant to NSSI among men, although this should be addressed by future research using more sophisticated measures of impulsivity and NSSI.

The weighted lifetime prevalence of NSSI in our sample was 2.69%, slightly lower than the 5.5% lifetime prevalence for adults reported in a more recent meta-analysis [32]. Prevalence estimates of NSSI vary considerably depending on methodological factors [32]. Lower prevalence estimates are associated with the use of adult samples, community-based rather than clinical or university-based samples, face-to-face interviews, NSSI measures with a yes/no format, and NSSI measures that do not specify multiple NSSI methods [32], all of which characterized the NPMS and could have accounted for the lower prevalence reported here. Therefore, it is not surprising that a recent study found a lifetime prevalence of 3.1% in a representative sample of the German population who responded to a household survey similar to the NPMS [33].

The clinical implication of these findings is that, although impulsivity has been proposed as a useful target for treating patients with NSSI [14], therapies directed at mood stabilization might be more clinically useful or at least more so than targeting impulsivity alone. A recent review found evidence that psychological interventions emphasizing emotional regulation such as dialectical behaviour therapy are effective at treating NSSI [34]. Medications such as lamotrigine and atypical antipsychotics reduce affective instability [35, 36] and could be useful for treating NSSI. Maintaining sleep and exercise may reduce affective instability [24], and could be useful preventative interventions for persons with affective instability at risk for NSSI.

The main strength of this study was the use of data from a large, nationally representative sample that allowed us to make longitudinal predictions over time. The disadvantage to this approach was that we were forced to rely upon categorical measures of affective instability and impulsivity when both traits can be better assessed with dimensional personality questionnaires [11, 17, 37]. Affective instability can also be measured prospectively via ecological momentary assessment [38, 39], avoiding the limitations associated with retrospective self-report questionnaires. On the other hand, the impulsivity item does correlate with corresponding facets from the Five-Factor model of personality [40], and single-item questions that assess affective instability also correlate well with ecological momentary assessment [38, 39]. Furthermore, it is is not feasible to use ecological momentary assessment in large epidemiological studies. Another disadvantage was that frequency and severity of NSSI was not assessed. NSSI can be a chronic problem with many episodes, although most people who engage in self-injury do so only once or a few times [2]. Interestingly, one study found no differences for impulsivity and emotional dysregulation between adolescents who engaged in NSSI once or multiple times [41]. Another strength is that we were also able to control for a diagnosis of borderline personality disorder, underscoring the effect of affective instability and impulsivity on NSSI in people without the disorder. The data is not recent, but our goal was to study the relationship between constructs, not estimate prevalences, and it is unlikely that the former would have changed over time. In addition, there are other forms of negative affect (e.g., anger) not considered in this study that could be included in future research. Affective instability is a broad construct involving rapid shifts between multiple emotional states (e.g., anger, sadness, fear [37]). The relationship between affective instability and impulsivity leading to NSSI will likely be more nuanced when instability in each emotional state is considered separately. Longitudinal designs are notably lacking in studies of NSSI [16]. Therefore, although our results should be viewed within the context of the aforementioned limitations, we consider them valuable nonetheless.

## Conclusions

Impulsivity and affective instability both predicted the onset of NSSI in a general population sample, whereas only affective instability predicted the continuation of NSSI. The relationship between impulsivity and NSSI is at least partially accounted for by affective instability. Clinical attention and research should focus on affective instability to advance our understanding of NSSI and enable more effective treatments to be developed for this condition.

## Abbreviations

CIS-R: Revised clinical interview schedule
DSM-IV: Diagnostic and statistical manual of mental disorders, 4th edition
NPMS: National psychiatric morbidity survey
NSSI: Nonsuicidal self-injury
SCID-II: Structured clinical interview for DSM-IV Axis II personality disorders
